# Effects of irrigation techniques on soil carbon sequestration and yield maintenance in non-rainfed croplands under straw return

**DOI:** 10.3389/fmicb.2026.1844595

**Published:** 2026-06-26

**Authors:** Wei Cheng, Juntao Cui, Bing Zhang, Long Ming

**Affiliations:** 1 College of Humanities & Information, Changchun University of Technology, Changchun, China; 2 College of Resources and Environmental, Jilin Agricultural University, Changchun, China; 3 College of Landscape Architecture, Changchun University, Changchun, China

**Keywords:** agricultural development, irrigation methods, maize yield, microorganisms, straw return

## Abstract

**Introduction:**

Driven by policies promoting green agricultural development and straw resource utilization, straw return has become an important strategy for improving cropland quality and enhancing farmland carbon sequestration. However, in non-rainfed croplands, stable crop production still depends on appropriate irrigation. Under straw return, the effects of different irrigation techniques on soil carbon sequestration and crop yield remain unclear, limiting the optimization of irrigation regimes and the synergistic enhancement of farmland carbon sequestration and yield stability.

**Methods:**

Based on this, the present study was conducted in a non-rainfed maize-growing region of Northeast China using high-throughput sequencing and statistical modeling. This study revealed how different irrigation techniques under straw return altered soil microbial communities, enzyme activities, and carbon components, thereby influencing crop yield.

**Results:**

Under the BICS treatment, microbial network structure and dissolved organic carbon were strongly associated with variations in maize yield. In contrast, the SDCS treatment increased particulate organic carbon content by 15.28% compared with BICS, and exhibited higher laccase activity (up to 2.38 nmol min^−1^ g^−1^), greater fungal richness, and a higher proportion of K-strategist microorganisms. Among these factors, particulate organic carbon accumulation and shifts in microbial life-history strategies may represent important drivers associated with yield improvement. The DPCS treatment exhibited the highest maize yield (14,011.90 kg hm^−2^), soil carbon component contents, sucrase activity, and bacterial richness, among which bacterial richness, laccase activity, and easily oxidizable organic carbon showed strong correlations with yield variation.

**Discussion:**

This study contributes to a deeper understanding of how irrigation techniques under straw return regulate soil carbon sequestration and crop yield formation, and provides a theoretical basis for optimizing water management strategies that jointly promote carbon sequestration and stable production in non-rainfed croplands.

## 1 Introduction

Driven by policies promoting green agricultural development and straw resource utilization, straw return has gradually become an important management practice for maintaining cropland quality and enhancing the carbon sink function of farmlands (Liu et al., 2023). Straw not only serves as an exogenous organic input that provides renewable carbon sources to the soil, thereby promoting nutrient cycling and the release of plant-available nutrients (Zhou R. et al., 2025), but also improves soil structure and enhances soil water-holding capacity, thereby contributing to soil moisture conservation (Xing et al., 2025). However, in non-rainfed croplands, precipitation is often insufficient to meet crop water demands during critical growth stages, making irrigation essential for maintaining stable crop production and sustaining farmland productivity (Rosa et al., 2020; Yang et al., 2026). Different irrigation techniques create distinct soil water supply patterns. Flood irrigation mainly increases soil moisture and wetting depth in the plow layer through surface water infiltration, whereas drip irrigation delivers water directly and quantitatively to the root zone, thereby reducing ineffective water loss and improving water-use efficiency (Zhang et al., 2022; Wang S. et al., 2026). Therefore, under straw return, irrigation techniques not only influence water supply processes, but may also alter the effects of straw on water retention, carbon input, and nutrient release, thereby affecting the synergistic relationship between soil carbon sequestration and stable crop production in non-rainfed croplands (Liu et al., 2024).

Previous studies have shown that the combination of straw return and irrigation can enhance crop yield (Wu L. et al., 2026), and that this effect is closely associated with the soil moisture regime, microbial activity, enzymatic reactions, and carbon fraction transformation. Straw return improves soil pore structure, promotes aggregate formation, enhances soil water-holding capacity, and provides relatively stable ecological niches for microorganisms (Zhao Y. et al., 2025). Meanwhile, microbial biomass carbon (MBC) and dissolved organic carbon (DOC) released during straw decomposition can stimulate the activities of lignin-degrading oxidative enzymes, such as polyphenol oxidase (S-PPO) and laccase (S-SL), thereby accelerating the mineralization of recalcitrant organic matter, carbon turnover, nutrient release, and soil fertility improvement (Wang X. et al., 2025). In this process, irrigation regulates soil water availability, aeration status, and thermal conditions, thereby alleviating the adverse effects of drought stress on crop growth (Liu and Wang, 2023; Chen et al., 2026; Ning et al., 2025), while also providing essential moisture conditions for straw decomposition and soil organic carbon turnover (Liu et al., 2026). Appropriate water supply can further reactivate dormant microbial communities in dry soils and promote the proliferation of organic carbon-dependent bacteria as well as functional microorganisms involved in nitrogen fixation and phosphorus solubilization (Zhang et al., 2025; Cui et al., 2025; Sun et al., 2021). Such changes in resource availability and environmental conditions may also alter microbial life-history strategies, leading to differential responses between r-strategist and K-strategist taxa (Li Z. et al., 2025). In addition, suitable soil moisture conditions can enhance the activities of metabolism-related enzymes, such as sucrase (S-SC) and catalase (S-CAT), thereby promoting carbohydrate hydrolysis and antioxidative reactions, improving soil nutrient availability, and increasing crop water- and fertilizer-use efficiency (Jing et al., 2025; Chi et al., 2026). Conversely, straw return can prolong water retention in the root zone after irrigation by improving soil structure and aggregate stability, reducing rapid water loss and thereby favoring microbial diversity maintenance and carbon transformation efficiency (Wang et al., 2024). However, different irrigation techniques exert distinct regulatory effects on soil water distribution, aeration conditions, and the root-zone environment (Lu et al., 2024; Yang et al., 2025). Flood irrigation wets the entire plow layer and can rapidly increase soil water content over the short term, but may also create temporary waterlogging or relatively anaerobic conditions in poorly drained areas, thereby influencing aerobic and anaerobic microbial activities as well as soil carbon cycling processes (Cheng et al., 2022). In contrast, drip irrigation creates a relatively moist and well-aerated microenvironment around the root zone, which is conducive to improving irrigation water-use efficiency and maintaining the activity of specific microbial communities (Zhang X. et al., 2024). Furthermore, shallow buried drip irrigation and mulched drip irrigation differ in terms of water infiltration position, evaporation suppression, and root-zone hydrothermal conditions, which may further affect straw decomposition, enzyme activities, carbon fraction transformation, and crop yield responses. As key biological drivers of soil carbon transformation and nutrient release, shifts in microbial community structure and function may represent a critical linkage among irrigation techniques, straw return, and crop yield formation (Fang et al., 2025).

The combination of straw return and irrigation has been widely recognized as an effective strategy for improving crop yield and soil quality. However, existing studies have primarily focused on the apparent effects of these practices on crop production or soil property improvement, whereas the synergistic relationships among soil carbon components, enzyme activities, and microbial communities during straw return under different irrigation techniques, as well as their differential regulatory effects on yield formation, remain poorly understood. This knowledge gap limits the synergistic optimization of straw return and irrigation regimes and constrains the development of carbon sequestration-oriented and yield-maintenance management strategies for non-rainfed croplands. To address this issue, a field experiment was conducted in a typical non-rainfed maize-growing region of Northeast China. Under straw return, three representative irrigation techniques, including flood irrigation, shallow buried drip irrigation, and mulched drip irrigation, were established to systematically evaluate their effects on soil carbon fraction accumulation, carbon-related enzyme activities, microbial community structure, and crop yield. In addition, the interactions among key soil ecological factors and their associations with yield formation were analyzed. We hypothesized that, under straw return, different irrigation techniques would generate distinct effects on carbon sequestration and yield stability by regulating microbial communities, enzyme activities, and carbon transformation processes. The findings of this study will deepen the understanding of how irrigation management under straw return influences soil carbon turnover and crop yield formation in non-rainfed croplands, and provide theoretical support and technical references for regional precision agriculture and soil health management.

## 2 Materials and methods

### 2.1 Site description

The study was conducted in a non-rainfed farmland in northern China. The tested soil type was meadow soil, and the experimental site was located in Ningjiang District, Songyuan City, Jilin Province (45°26′N, 126°28′E). The regional climate was classified as a continental monsoon climate, characterized by cold winters and hot summers. The average annual rainfall was 350 mm, with most precipitation occurring between May and October. The basic soil properties were as follows: silt, sand, and clay contents accounted for 42.41, 38.39, and 19.20%, respectively. Soil organic carbon, alkali-hydrolyzable nitrogen, available phosphorus, and available potassium contents were 17.06 g kg^−1^, 84.02 mg kg^−1^, 16.77 mg kg^−1^, and 64.24 mg kg^−1^, respectively, with a soil pH of 8.0. Detailed meteorological data during the crop growing season are presented in Supplementary Figure S1.

### 2.2 Experimental design and arrangement

The microplot experiment was conducted in a maize-growing region during 2021 and 2022. Three irrigation treatments were established: flood irrigation (BICS), shallow buried drip irrigation (SDCS), and mulched drip irrigation (DPCS). Three plots were established for each treatment, with each plot covering an area of 5 m × 10 m (50 m^2^). Within each plot, soil samples were collected from five sampling points and composited into a single representative sample to balance field heterogeneity and sampling practicality. All treatments were conducted under straw return conditions. The maize variety used in the experiment was “Fumin 985,” with a planting density of 70,000 plants per hectare.

A wide–narrow row planting pattern was adopted, with wide rows spaced at 80 cm and narrow rows at 40 cm. The sowing depth was 4–5 cm. Sowing was performed using a modified integrated seeder (2BMZ-6; First Tractor Group Co., Ltd., Luoyang, China), which simultaneously applied basal fertilizer, sowed seeds, covered soil, compacted the soil surface, laid plastic film, and installed drip irrigation pipes.

After harvest in the previous season, maize straw was mechanically chopped into 1–2 cm pieces and evenly distributed across the soil surface 10 days before spring sowing. The straw was then incorporated into the 15 cm soil layer by rotary tillage. The straw used in 2021 was derived from maize harvested from the same experimental field in 2020, whereas the straw used in 2022 originated from maize harvested in 2021. Straw was returned at the full retention rate (13,600 kg hm ^−2^).

In the DPCS treatment, plastic film with a thickness of 0.012 mm and a width of 800 mm was used at an application rate of approximately 45 kg hm^−2^. Independent drip irrigation units consisted of drip tapes (8,000 m hm^−2^), Φ63 PE main pipes (75 m hm^−2^), and associated fittings. A drip tape was installed at the center of each narrow row, with emitter spacing set at 25 cm. In addition, a fertilization tank (ZZYP-16C DN50; Shanghai Chuanhu Valve Co., Ltd., China) with a capacity of 30 t h^−1^ was equipped for fertigation. In contrast, the SDCS treatment used shallow buried drip irrigation pipes without plastic film mulching. The irrigation configuration of the SDCS treatment was otherwise similar to that of the DPCS treatment. The BICS treatment did not use drip irrigation or plastic film, but instead adopted conventional pipe irrigation.

Apart from the irrigation techniques, all other field management practices remained consistent across treatments. The BICS, DPCS, and SDCS treatments all adopted a basal fertilizer-topdressing management regime. Basal fertilizer was applied before sowing using a compound fertilizer (N-P-K: 15–15-15) at a rate of 450 kg hm^−2^. The fertilizer was evenly broadcast onto the prepared soil surface and subsequently incorporated into the topsoil through shallow tillage or rotary cultivation. Urea fertilizer (46% N) was used for topdressing at the jointing stage, trumpet stage, and pre-tasseling stage, with 30 kg hm^−2^ applied at each stage. In the BICS treatment, topdressing fertilizer was manually applied to the soil, while irrigation was conducted simultaneously with the DPCS and SDCS treatments. In the DPCS and SDCS treatments, topdressing was applied through fertigation. Specifically, clean water was first supplied for approximately 30 min to pre-wet the soil, followed by the continuous application of dissolved urea through the irrigation system for approximately 2 h, and finally flushed with clean water for another 30 min to ensure uniform fertilizer distribution. The total irrigation amount and fertilizer input were maintained consistently across all treatments to ensure comparability among treatments, with a total irrigation amount of 240 mm per hectare during the growing season. The irrigation source was groundwater, which showed no presence of heavy metals or harmful microorganisms.

### 2.3 Soil sample collection

For each plot, five sampling points were randomly selected while avoiding edge effects. The samples were then composited to represent plot-level soil conditions. Soil samples were collected in April and September 2021 and in September 2022. For both the BICS and drip irrigation treatments (SDCS and DPCS), soil samples were collected approximately 10 cm away from the water source to represent the root-zone soil most strongly influenced by water and fertilizer inputs. Soil samples were collected from the 0–20 cm layer using clean stainless steel soil augers. After collection, crop residues, roots, stones, and other impurities were immediately removed. The samples were then placed into clean self-sealing bags, labeled, and sealed for storage. Upon returning to the laboratory, the soil samples were divided into three portions according to analytical objectives. One portion was air-dried at room temperature, cleaned of impurities, ground, and passed through a 2 mm sieve for soil physicochemical analysis. A second portion was stored at 4 °C for the determination of enzyme activities and MBC. The remaining portion was stored at −80 °C for soil DNA extraction and molecular biological analyses.

### 2.4 Determination of soil properties

Soil measurements were conducted according to the sampling schedule. Soil samples collected in September 2021 and 2022 were used for soil carbon fraction analysis, whereas samples collected in September 2022 were used for enzyme activity determination and microbial community DNA analysis.

Soil organic carbon (SOC) was determined using the potassium dichromate-sulfuric acid oxidation method with external heating, following previously reported procedures (Li Y. et al., 2025). MBC was measured using the chloroform fumigation–potassium sulfate extraction method based on a correction factor (Zhou J. et al., 2025). DOC was extracted with water, filtered, and quantified using a TOC analyzer (Davenport et al., 2023). Easily oxidizable organic carbon (EOC) was determined using the potassium permanganate oxidation method, followed by spectrophotometric measurement at 565 nm (Huang, 2021). Particulate organic carbon (POC) was determined by dispersing soil samples with sodium pyrophosphate, sieving them through a 0.053 mm mesh, and measuring the organic carbon content in particles larger than 0.053 mm (Wang Y. et al., 2026).

Soil enzyme activities were determined using microplate assay methods with commercial assay kits purchased from Lemore Biological Technology Co., Ltd. (Shanghai, China). All procedures were performed strictly according to the manufacturer’s instructions and validated using standard calibration curves. S-PPO was determined using L-DOPA as the substrate in a 50 mM sodium acetate buffer solution (pH 5.0). Samples were incubated at 25 °C with shaking for 1 h. The reaction was terminated in an ice bath, followed by centrifugation, and the absorbance of the supernatant was measured at 460 nm. Enzyme activity was calculated based on the production of red quinone compounds and expressed as μmol h^−1^ g^−1^ (Wang N. et al., 2022). S-SL was determined using ABTS as the substrate in a citrate–phosphate buffer system. Samples were incubated at 37 °C with shaking for 10 min. After termination in an ice bath and centrifugation, the absorbance of the supernatant was measured at 420 nm. Enzyme activity was calculated according to the generation rate of ABTS radicals and expressed as nmol min^−1^ g^−1^ (Zhang et al., 2021). S-SC was measured using sucrose as the substrate in a phosphate buffer solution (pH 5.5). Samples were incubated at 37 °C for 24 h. After incubation, DNS reagent was added, and the mixture was heated in a 92 °C water bath for 5 min for color development. Following cooling and dilution, absorbance was measured at 510 nm. Enzyme activity was calculated based on reducing sugar production and expressed as mg d^−1^ g^−1^ (Zhao et al., 2022). S-CAT was determined using hydrogen peroxide as the substrate. Samples were incubated at 25 °C with shaking for 20 min, followed by centrifugation, and absorbance was measured at 240 nm. Enzyme activity was calculated based on hydrogen peroxide consumption and expressed as mmol d^−1^ g^−1^ (Wang S. et al., 2022).

### 2.5 Soil DNA extraction, sequencing, and analysis

Total soil microbial DNA was extracted from fresh soil samples using the MagBeads FastDNA Kit for Soil (MP Biomedicals, CA, USA) according to the manufacturer’s instructions. DNA integrity was assessed by 0.8% agarose gel electrophoresis, while DNA concentration and purity were determined using a NanoDrop UV spectrophotometer (Thermo Scientific, NC2000).

Bacterial community analysis targeted the hypervariable V3-V4 regions (~480 bp) of the 16S rRNA gene using the primer pair 338F (5′-barcode-ACTCCTACGGGAGGCAGCA-3′) and 806R (5′-GGACTACHVGGGTWTCTAAT-3′) for PCR amplification. The total volume of the PCR reaction system was 20 μL, consisting of 0.25 μL Q5 High-Fidelity DNA Polymerase, 5 μL 5 × Reaction Buffer, 5 μL 5 × High GC Buffer, 2 μL dNTPs (10 mM), 2 μL template DNA, 1 μL forward primer (10 μM), 1 μL reverse primer (10 μM), and 8.75 μL nuclease-free water. The PCR amplification program was as follows: initial denaturation at 98 °C for 5 min, followed by 25 cycles of denaturation at 98 °C for 30 s, annealing at 52 °C for 30 s, and extension at 72 °C for 45 s, with a final extension at 72 °C for 5 min and subsequent storage at 12 °C. Fungal community analysis targeted the ITS gene V1 region (approximately 280–500 bp), with primers ITS5 (5′-GGAAGTAAAAGTCGTAACAAGG-3′) and ITS2 (5′-GCTGCGTTCTTCATCGATGC-3′) for amplification. The total volume of the PCR reaction system was 20 μL, consisting of 0.25 μL Q5 High-Fidelity DNA Polymerase, 5 μL 5 × Reaction Buffer, 5 μL 5 × High GC Buffer, 2 μL dNTPs (10 mM), 2 μL template DNA, 1 μL forward primer (10 μM), 1 μL reverse primer (10 μM), and 8.75 μL nuclease-free water. The PCR amplification program consisted of an initial denaturation at 98 °C for 5 min, followed by 30 cycles of denaturation at 98 °C for 30 s, annealing at 55 °C for 45 s, and extension at 72 °C for 45 s, with a final extension at 72 °C for 5 min and subsequent storage at 12 °C. Each sample was amplified in triplicate. The PCR products were examined by 2% agarose gel electrophoresis, and the target bands were excised and purified using the Axygen Gel Extraction Kit. The purified products were subsequently used for bacterial and fungal library construction and high-throughput sequencing analysis. Library construction and Illumina high-throughput sequencing were conducted by Aovisen Technology Co., Ltd.

The raw sequencing data were processed using QIIME2 software (v2022.11). Sequences were first demultiplexed using the demux plugin, followed by primer trimming and removal of unmatched reads using the cutadapt plugin. Denoising, quality filtering, paired-end merging, and chimera removal were performed using the DADA2 plugin to generate amplicon sequence variants (ASVs) and their abundance tables. The ASV tables were subsequently merged after individual sample processing, and singleton ASVs were removed. The number of valid sequencing reads (clean tags) for fungal and bacterial samples ranged from 37,903 to 112,212 and from 46,334 to 160,463, respectively. To ensure comparability in Alpha-diversity analysis, all samples were rarefied to the minimum sequencing depth prior to analysis. The rarefaction curves demonstrated that the minimum sequencing depth sufficiently covered the major microbial diversity present in the samples, thereby providing adequate support for downstream diversity and community composition analyses (Supplementary Figure S2). To minimize the effects of sequencing depth variation among samples on Beta-diversity and community composition analyses, the ASV table was normalized based on total sequence counts. Negative control samples were included throughout the experimental process to detect potential contamination, and low-abundance ASVs identified in blank controls were removed to ensure data reliability. The processed ASV table was subsequently used for all downstream analyses, including Alpha-diversity, Beta-diversity (PCA), community composition, and network analysis. Sequence length distribution statistics were performed using R software (v4.3.0).

Taxonomic classification was performed using the RDP Classifier (v2.2).^1^ Bacterial 16S rRNA gene sequences were aligned against the SILVA database (Release 138.1), whereas fungal ITS sequences were aligned against the UNITE database (Release 8.0).

### 2.6 Statistical analysis

The statistical significance among treatments was assessed by analysis of variance (ANOVA), followed by Tukey HSD *post hoc* test, with *p* < 0.05 considered significant. All statistical analyses were performed using IBM SPSS Statistics v25.0 (Li et al., 2023). Bar plots of maize yield, SOC, and soil carbon components (DOC, EOC, POC, and MBC) were generated using OriginPro 2021. Boxplots of carbon-related enzyme activities (S-PPO, S-CAT, S-SL, and S-SC), microbial alpha-diversity indices (Chao1 and Shannon), and stacked bar plots of microbial community composition were generated using the ggplot2 package in R (v4.3.3).

Microbial community Beta-diversity analysis was performed using the vegan package in R (v4.3.3). Principal component analysis (PCA) was conducted to evaluate differences in community structure, and the significance of treatment effects was assessed using analysis of similarities (ANOSIM). The classification of microbial taxa into r-strategists and K-strategists was determined based on published literature and statistical analyses, with detailed results provided in Supplementary Table S1. The classification results were subsequently used for community functional analysis and ecological strategy comparisons. Molecular ecological networks (MEN) were constructed based on random matrix theory (RMT) to elucidate ecological interactions among treatments (Raimi et al., 2023). Briefly, the ASV abundance table was first normalized, after which pairwise Spearman correlation coefficients among ASVs were calculated and tested for significance (*p* < 0.05). The network threshold (rc) was automatically determined using the rm.get.threshold function in R, which identifies the minimum non-random correlation coefficient based on RMT and is used to retain robust network edges. Only significant correlations with coefficients ≥ rc were retained to construct the final MEN (Zhang B. et al., 2024). Visualization of the MEN and calculation of network topological properties, including node and edge numbers, were performed using Gephi software. In addition, network cohesion, including positive cohesion, negative cohesion, and total cohesion, was calculated to quantify the internal connectivity of microbial communities and was presented using boxplots. Negative cohesion has been associated with lower community compositional turnover and can therefore provide insights into community stability (Herren and McMahon, 2017; Ban et al., 2026).

Structural equation modeling (SEM) was performed using the piecewiseSEM package in R (v4.3.3). Linear regression submodels were constructed based on crop yield, soil carbon, microbial, and enzyme activity data from different treatments and subsequently integrated into a comprehensive model to characterize the direct and indirect relationships among variables (Dietrich et al., 2023). Model fit was evaluated using Fisher’s C statistic and its corresponding *p* value. A *p* value > 0.05 indicated that the conditional independence claims corresponding to omitted paths were not significant, suggesting that the model adequately fitted the observed data. Model robustness was further evaluated based on degrees of freedom (df) and the significance of path coefficients. These procedures ensured the robustness and reliability of the path coefficients for elucidating the potential mechanisms through which different treatments influenced crop yield and soil carbon dynamics. Random forest (RF) analysis was conducted using the randomForest package in R (v4.3.3) to evaluate the explanatory contributions of endogenous variables in the SEM to crop yield (Yu et al., 2019). A random seed was set (set.seed(123)) to ensure reproducibility. The model included all predictor variables and was performed with 50 repeated runs combined with 10-fold cross-validation to improve the robustness and reliability of the results. Variable importance values (importance = TRUE) were extracted and visualized using the ggplot2 package (Sun et al., 2025).

## 3 Results

### 3.1 Effects of different irrigation techniques under straw return on maize yield and soil carbon components

**Figure 1:**
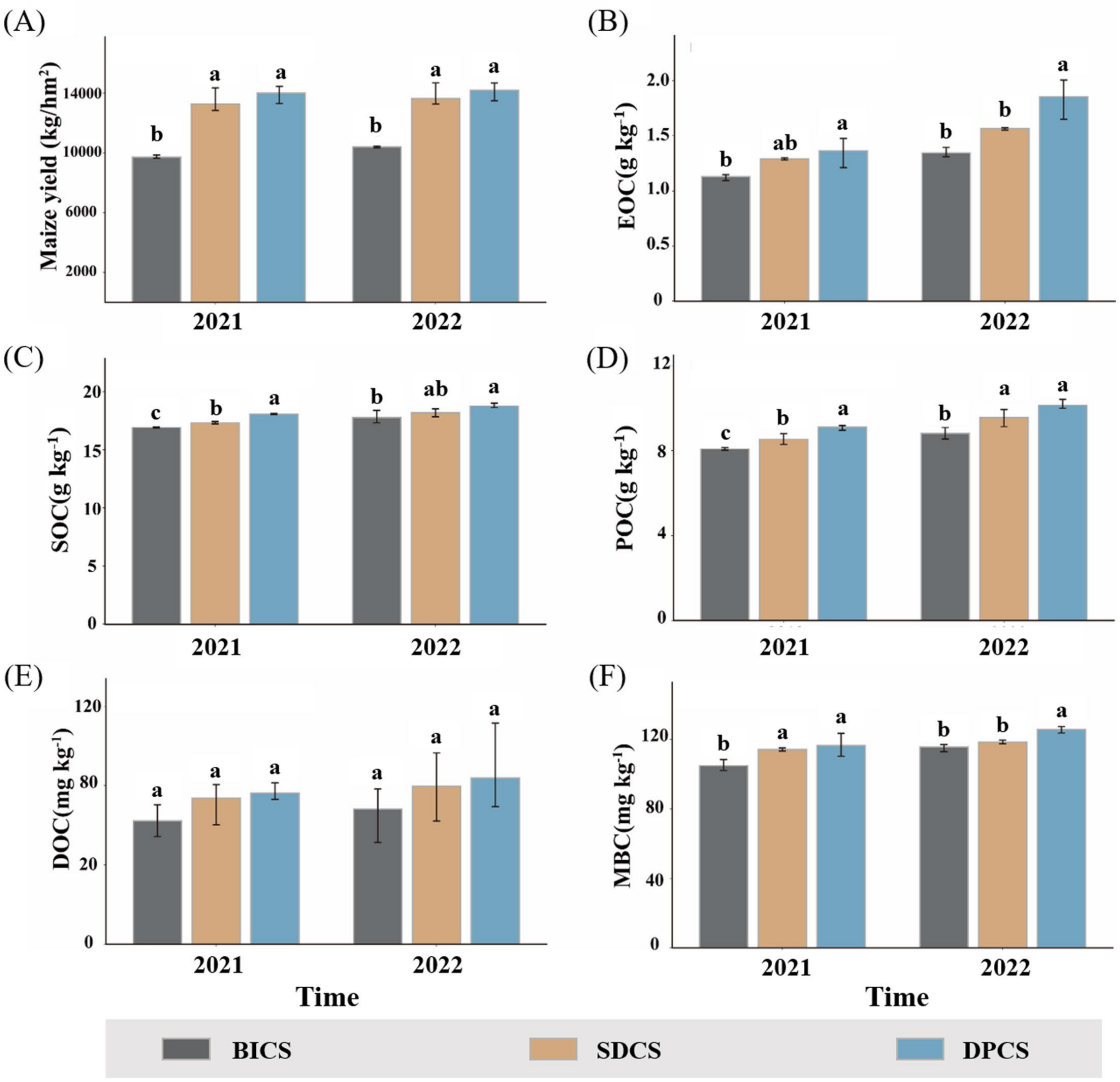
Maize yield, soil organic carbon (SOC), and soil carbon component contents under different treatments in 2021 and 2022. **(A)** Maize yield; **(B)** easily oxidizable organic carbon (EOC); **(C)** SOC; **(D)** particulate organic carbon (POC); **(E)** dissolved organic carbon (DOC); and **(F)** microbial biomass carbon (MBC). The data is expressed as mean ± standard error (*n* = 3). Significant differences between means at *p* < 0.05 in the Tukey HSD test are denoted by different lower-case letters.

Analysis of maize yield in 2021 and 2022 (Figure 1) showed that the SDCS and DPCS treatments significantly increased maize yield compared with the BICS treatment. In 2021, maize yield increased by 35.87 and 31.00% under the DPCS and SDCS treatments, respectively, relative to BICS. In 2022, the corresponding increases were 39.74 and 34.62%, respectively. Analysis of SOC and its carbon components revealed significant differences in SOC, EOC, POC, and MBC among irrigation treatments (*p* < 0.05), whereas DOC showed no significant difference. Under the BICS treatment, SOC and all measured carbon components were generally lower than those under the SDCS and DPCS treatments. Carbon fraction contents under DPCS were consistently higher than those under BICS across both years. In addition, POC content under SDCS was significantly higher than that under BICS, with increases of 14.97 and 15.58% in 2021 and 2022, respectively. Overall, the DPCS treatment showed the strongest effects on maize yield enhancement and soil carbon accumulation, followed by SDCS, whereas the BICS treatment exhibited the weakest effects.

### 3.2 Effects of different irrigation techniques under straw return on soil carbon transformation-related enzyme activities

**Figure 2:**
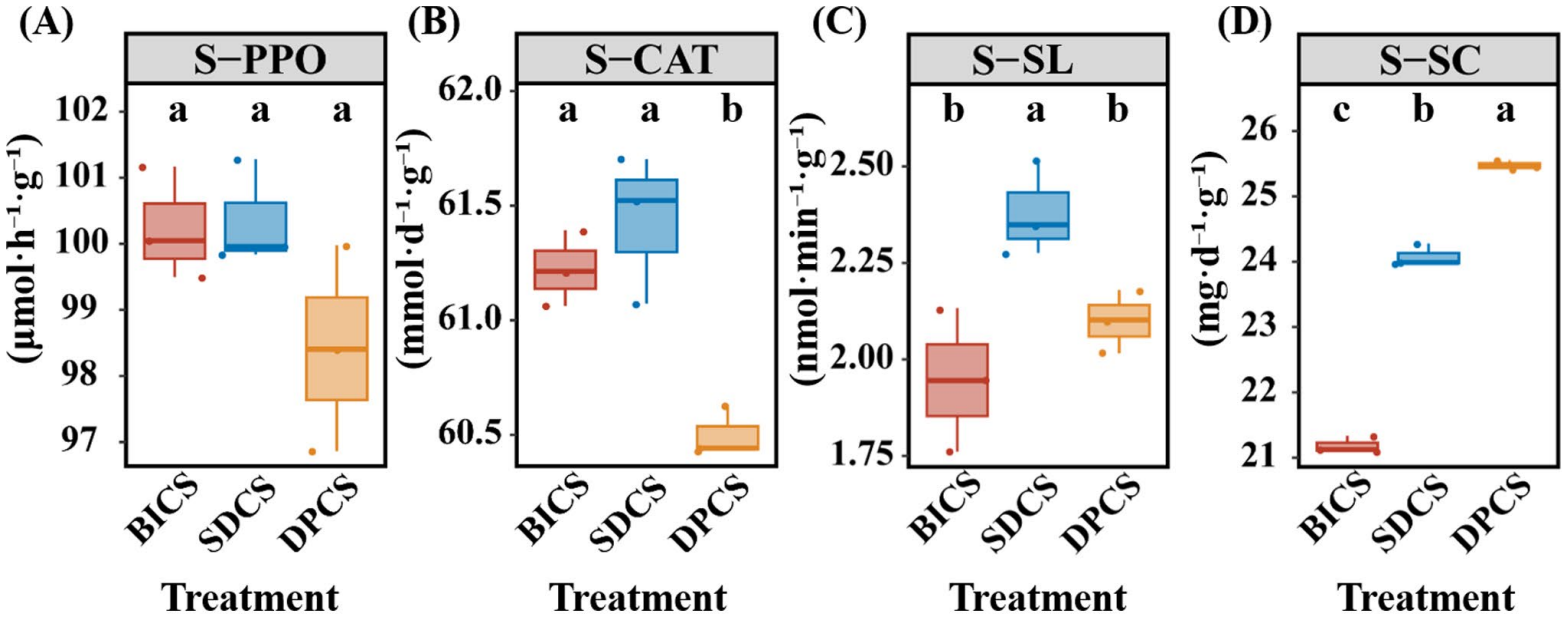
Carbon transformation-related enzyme activities under different treatments. **(A)** Polyphenol oxidase (S-PPO); **(B)** Catalase (S-CAT); **(C)** Laccase (S-SL); and **(D)** Sucrase (S-SC). Boxplots show the median, interquartile range, and minimum–maximum values (*n* = 3). Significant differences between means at *p* < 0.05 in the Tukey HSD test are denoted by different lower-case letters.

In this study, the activities of four enzymes involved in soil carbon transformation were analyzed under different irrigation treatments with straw return. The results showed significant differences in the activities of S-CAT, S-SL, and S-SC among treatments (*p* < 0.05), whereas no significant difference was observed for S-PPO (Figure 2). S-CAT activity was significantly higher under the BICS and SDCS treatments than under the DPCS treatment, with increases of 1.19 and 1.54%, respectively. S-SL activity was significantly higher under the SDCS treatment than under the DPCS and BICS treatments, with a maximum increase of 22.28%. In contrast, S-SC activity was significantly higher under the DPCS treatment than under the SDCS treatment, with an increase of 5.81%. Moreover, the SDCS treatment also showed significantly higher S-SC activity than the BICS treatment, with an increase of 13.67%.

### 3.3 Effects of different irrigation techniques under straw return on soil microbial community diversity and structure

**Figure 3:**
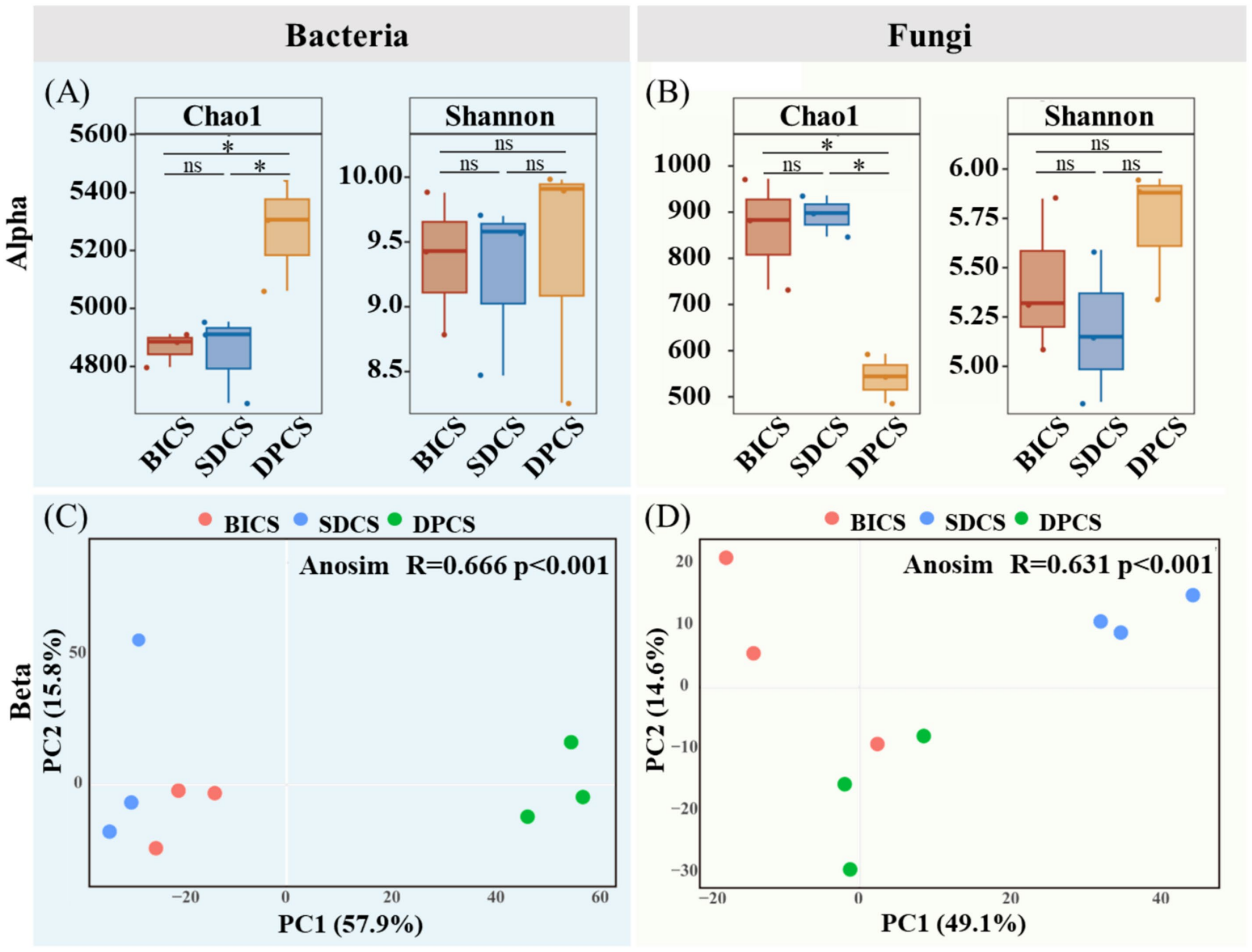
Soil microbial Alpha and Beta diversity under different treatments. **(A)** Bacterial Alpha diversity index; **(B)** fungal Alpha diversity index; **(C)** bacterial Beta diversity index; **(D)** fungal Beta diversity index. Boxplots show the median, interquartile range, and minimum–maximum values (*n* = 3). Principal component analysis (PCA) of soil microbial communities under different treatments. Each point represents a sample (*n* = 3). Asterisks denote for significant probability levels (ns, *p* ≥ 0.05; *p < 0.05; **p < 0.01; ***p < 0.001).

In this study, the Chao1 and Shannon indices were used to evaluate the richness and diversity of microbial communities (Figure 3). The results showed significant differences in microbial community richness among irrigation treatments under straw return (*p* < 0.05), whereas no significant differences were observed in microbial diversity. Compared with the BICS and SDCS treatments, the DPCS treatment significantly increased the Chao1 index of the bacterial community by 8.36 and 8.77%, respectively. In contrast, the DPCS treatment significantly decreased the Chao1 index of the fungal community by 37.24 and 39.44%, respectively.

Microbial community Beta-diversity was assessed using PCA (Figure 3). The results showed clear separation of microbial samples among treatments in the two-dimensional ordination space. For the bacterial community, the DPCS treatment was clearly separated from the BICS and SDCS treatments along the PC1 axis. For the fungal community, the SDCS treatment was distinctly separated from the BICS and DPCS treatments along the PC1 axis. ANOSIM analysis further confirmed that the structural differences in both bacterial and fungal communities among treatments were statistically significant (*p* < 0.001).

### 3.4 Effects of different irrigation techniques under straw return on soil microbial community composition

**Figure 4:**
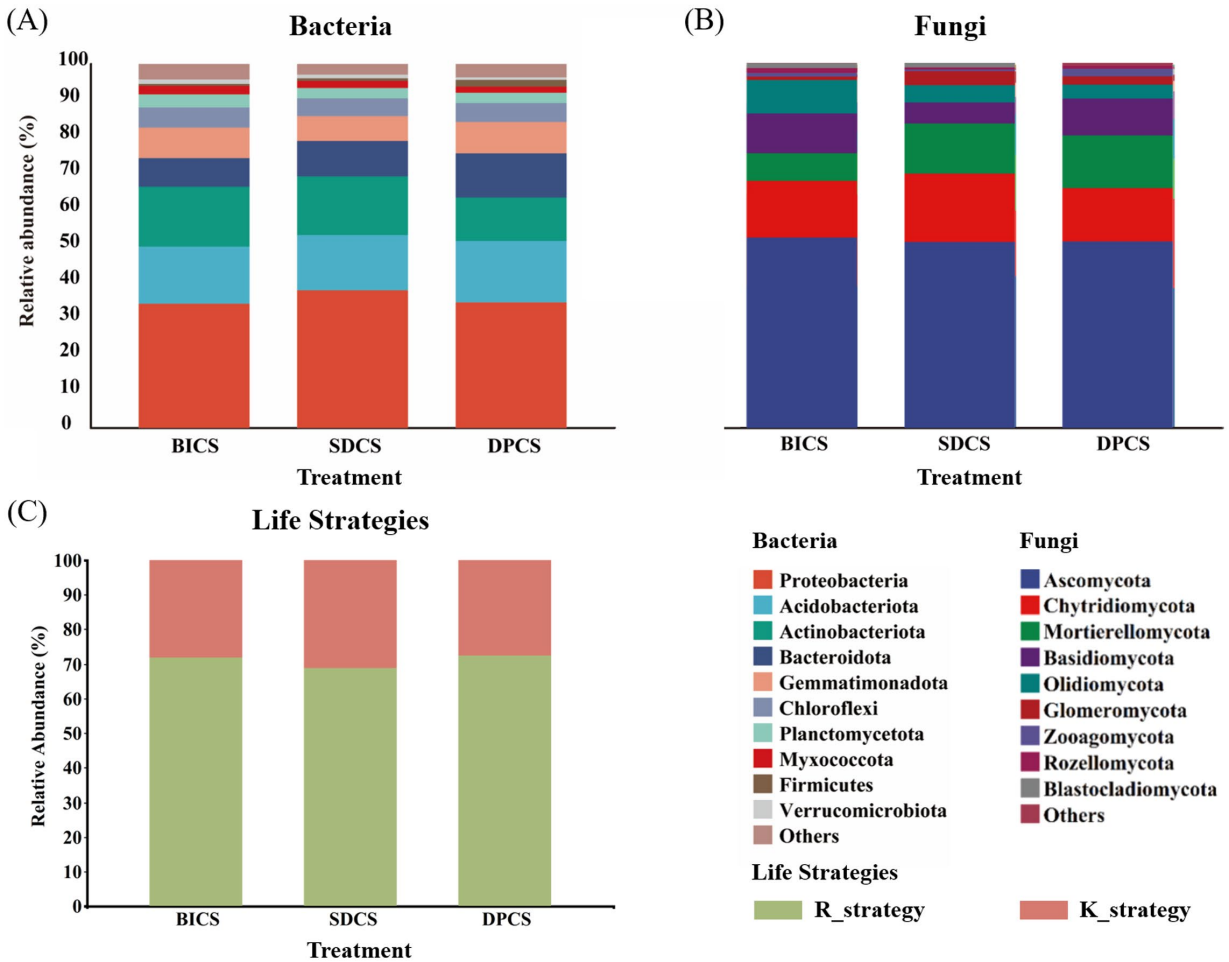
Soil microbial community composition and life-history strategies under different treatments. **(A)** Bacterial community composition; **(B)** Fungal community composition; and **(C)** Microbial life-history strategies. Data are based on three independent replicates (*n* = 3).

Based on phylum-level classification of the high-throughput sequencing data, Proteobacteria, Acidobacteriota, and Actinobacteriota were identified as the dominant bacterial phyla, collectively accounting for more than 60% of the bacterial community, with relatively stable abundances across treatments (Figure 4). Among them, Proteobacteria showed the highest relative abundance under the SDCS treatment, reaching 37.82%. In the fungal community, Ascomycota, Chytridiomycota, and Mortierellomycota were the dominant phyla, collectively accounting for more than 70% of the fungal community. The SDCS treatment exhibited a relatively higher proportion of Chytridiomycota (18.73%), whereas Mortierellomycota showed a higher relative abundance under the DPCS treatment (14.61%). In contrast, Ascomycota reached the highest relative abundance under the BICS treatment, accounting for 52.18%.

Analysis of microbial life-history strategies under different treatments showed that all treatments were predominantly dominated by r-strategist microorganisms. Among them, the DPCS treatment exhibited the highest proportion of r-strategist microorganisms, accounting for 72.28%, whereas the SDCS treatment showed the highest proportion of K-strategist microorganisms, reaching 31.28%.

### 3.5 Effects of different irrigation techniques under straw return on soil microbial networks

**Figure 5:**
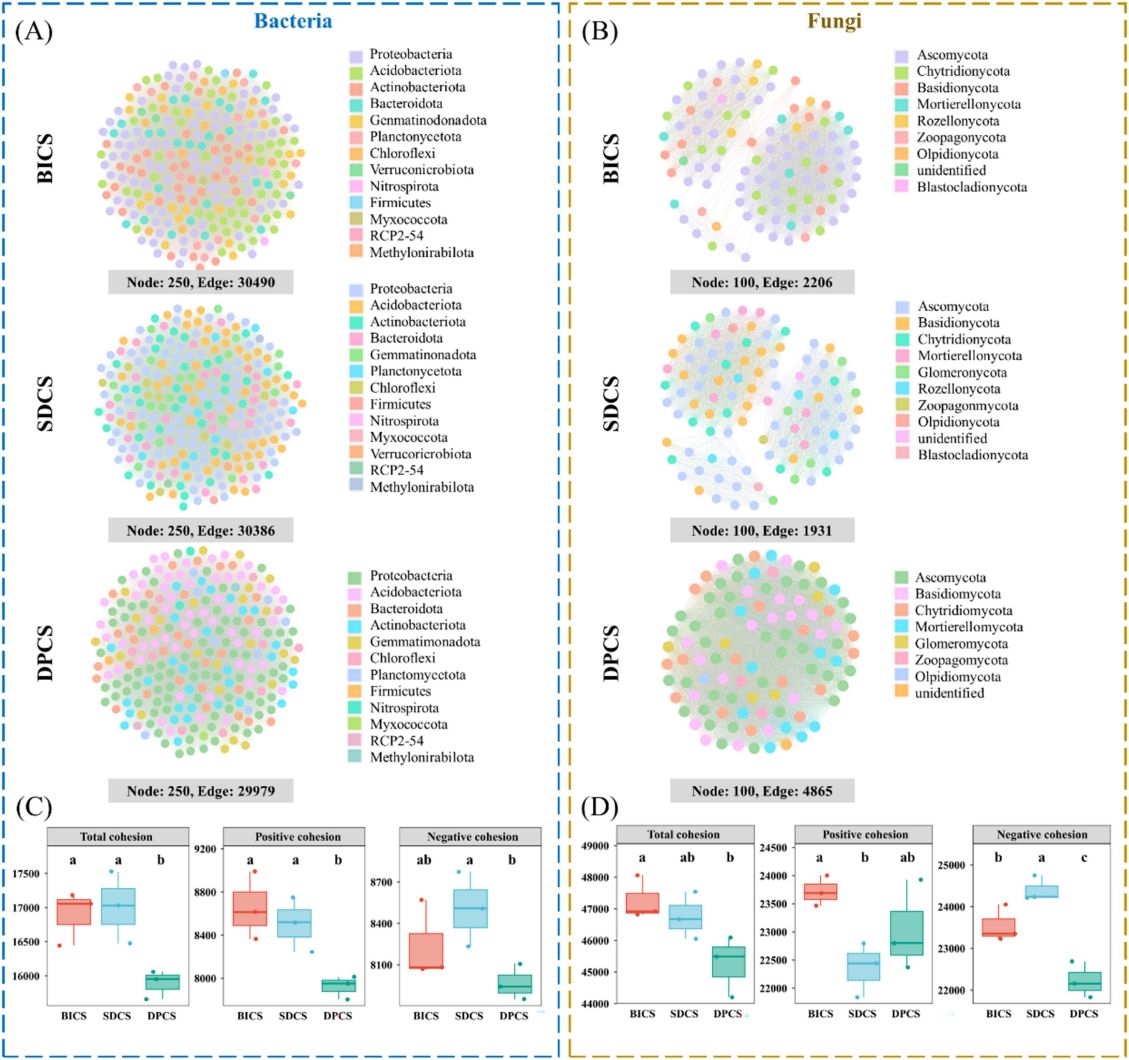
Soil microbial network structure and cohesion under different treatments (*n* = 3). **(A)** Bacterial network structure; **(B)** fungal network structure; **(C)** bacterial network cohesion; and **(D)** fungal network cohesion. Boxplots show the median, interquartile range, and minimum–maximum values (*n* = 3). Significant differences between means at *p* < 0.05 in the Tukey HSD test are denoted by different lower-case letters.

The significant differences in bacterial and fungal network topology were observed among treatments in the constructed molecular ecological networks (Figure 5). In the bacterial network, the BICS treatment exhibited a greater number of species connections, whereas in the fungal network, the DPCS treatment showed the highest number of connections.

To evaluate microbial network connectivity and interaction strength under different treatments, network cohesion indices were calculated (Figure 5). The results showed significant differences in total cohesion, positive cohesion, and negative cohesion among treatments (*p* < 0.05). In the bacterial network, both total cohesion and positive cohesion were significantly higher under the BICS and SDCS treatments than under the DPCS treatment, with increases of 6.32 and 9.24% for total cohesion, and 7.35 and 7.06% for positive cohesion, respectively. The SDCS treatment exhibited the highest negative cohesion, reaching 8505.33. In the fungal network, the BICS treatment showed the highest total cohesion (47,265.24) and positive cohesion (23,719.30). However, the SDCS treatment exhibited the highest negative cohesion, which was significantly higher than those under the BICS and DPCS treatments by 3.62 and 9.78%, respectively.

### 3.6 Pathway analysis of yield enhancement and soil fertility regulation under different irrigation techniques with straw return

**Figure 6:**
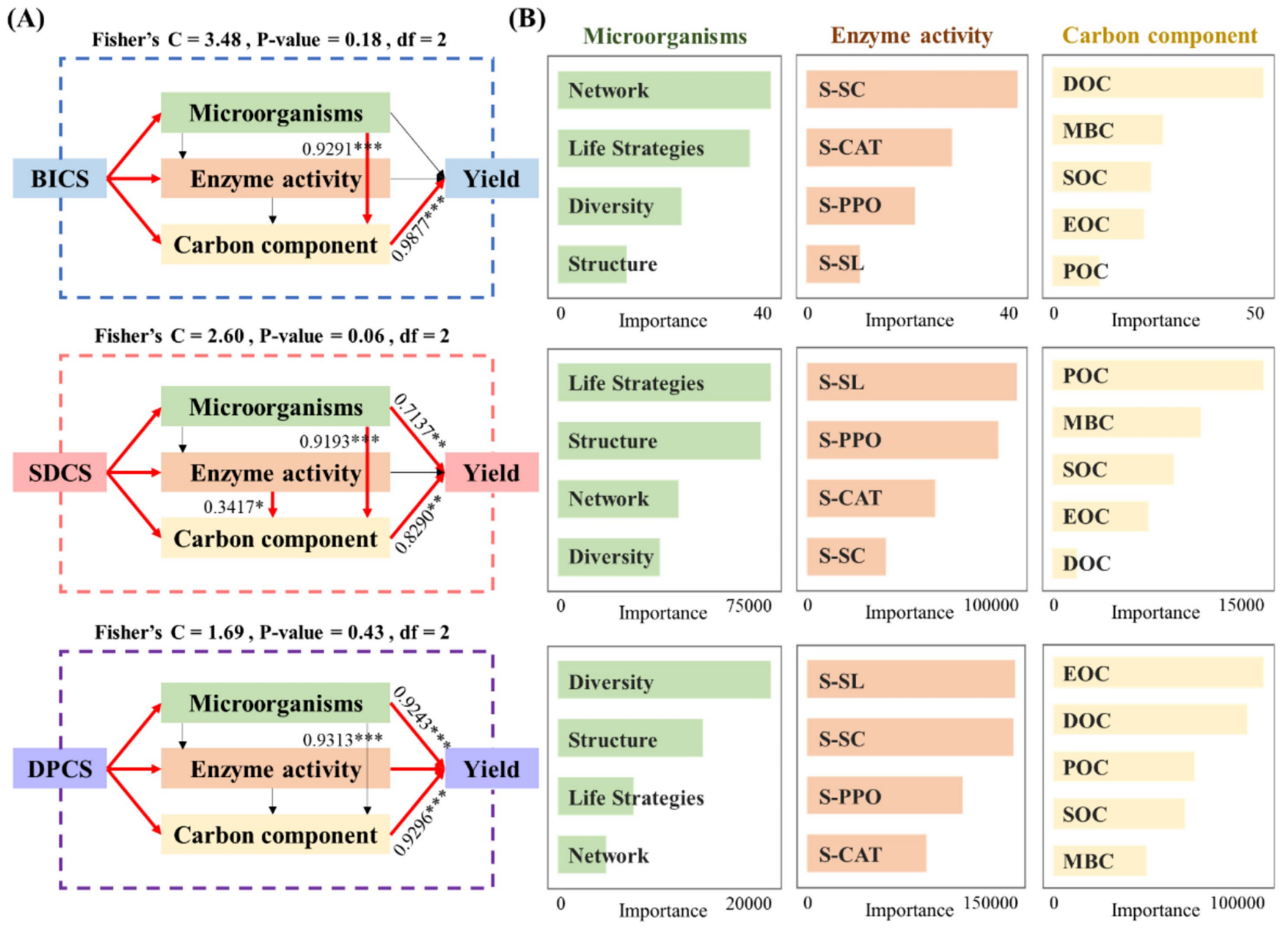
Pathways of yield enhancement and soil fertility improvement and key contributing factors under different treatments. **(A)** SEM analysis; **(B)** RF analysis. Data are based on three independent replicates (*n* = 3). Asterisks denote for significant probability levels (*p < 0.05; **p < 0.01; ***p < 0.001).

Different irrigation techniques under straw return significantly affected maize yield, soil carbon-related components, carbon transformation-related enzyme activities, and microbial communities (Figure 6). To further elucidate the regulatory pathways underlying soil fertility improvement and yield enhancement, a SEM was constructed. Under the BICS treatment, the microbial community exerted a significant positive effect on soil carbon-related components (path coefficient = 0.9291, *p* < 0.001), which subsequently promoted maize yield (path coefficient = 0.9877, *p* < 0.001). Under the SDCS treatment, both the microbial community (path coefficient = 0.9139, *p* < 0.01) and carbon transformation-related enzyme activities (path coefficient = 0.3417, *p* < 0.05) jointly influenced soil carbon-related components, which in turn positively affected maize yield (path coefficient = 0.8290, *p* < 0.01). In addition, the microbial community directly promoted yield improvement (path coefficient = 0.7137, *p* < 0.01). Under the DPCS treatment, the microbial community (path coefficient = 0.9243, *p* < 0.001), carbon transformation-related enzyme activities (path coefficient = 0.9313, *p* < 0.001), and soil carbon-related components (path coefficient = 0.9296, *p* < 0.001) all exerted direct positive effects on maize yield, whereas interactions among these factors were relatively weak.

At the same time, the contribution of each endogenous variable to maize yield in the SEM was evaluated using a random forest model. The results showed that the dominant factors differed among treatments. Under the BICS treatment, the main contributing microbial factor was network structure, the primary enzyme factor was S-SC activity, and the key soil carbon-related component was DOC. Under the SDCS treatment, microbial life-history strategy was the dominant microbial factor, S-SL activity was the primary enzyme factor, and POC was the main soil carbon-related component. Under the DPCS treatment, microbial diversity was the dominant microbial factor, S-SL activity was the primary enzyme factor, and EOC was the key soil carbon-related component.

## 4 Discussion

### 4.1 Responses of soil carbon accumulation and enzyme activities to different irrigation techniques under straw return

This study investigated how flood irrigation, shallow buried drip irrigation, and mulched drip irrigation under straw return affected soil carbon component contents and carbon transformation-related enzyme activities. Although the three irrigation techniques received the same irrigation amount, differences in water delivery position and infiltration patterns among irrigation methods may place straw decomposition and carbon transformation processes under distinct soil environmental conditions, thereby leading to differences in soil carbon accumulation and transformation efficiency. The DPCS and SDCS treatments showed clear advantages in promoting soil carbon accumulation and carbon transformation-related enzyme activities. In particular, the DPCS treatment significantly increased SOC and related soil carbon component contents compared with the BICS treatment. This may be attributed to the lower surface disturbance and stronger soil moisture conservation effect, which are conducive to the transformation and retention of straw-derived carbon in the soil, thereby promoting soil organic carbon accumulation (Iqbal et al., 2020). In 2021, POC content under the SDCS treatment was 14.97% higher than that under the BICS treatment, while in 2022, the increase reached 15.58%. These results suggest that the SDCS treatment provided a more uniform and precise water supply (Cheng et al., 2025), thereby reducing water flow and surface erosion and promoting POC accumulation. In contrast, the BICS treatment caused greater fluctuations in soil moisture, which may have reduced the stability of the soil microenvironment and resulted in a weaker carbon accumulation effect (Guo and Li, 2024). In terms of enzyme activities, higher S-CAT activity was observed under the BICS and SDCS treatments, suggesting that different irrigation techniques influenced soil microbial metabolic processes and oxidative stress-related enzyme activities (Yang et al., 2023). The relatively high S-SL activity under the SDCS treatment may be associated with a water supply position that was more favorable for microbial growth, thereby enhancing straw decomposition and the transformation of complex carbon substrates (e.g., lignin) in the root zone (Lin et al., 2025). The DPCS treatment exhibited the highest S-SC activity, which may be attributed to film mulching reducing surface disturbance and promoting the preservation of labile carbon substrates (Sun and Han, 2025), thereby stimulating sucrase-mediated carbohydrate transformation processes (Sun et al., 2024). In contrast, no significant differences in S-PPO activity were observed among the three irrigation techniques, indicating that S-PPO may exhibit relatively low sensitivity to irrigation-induced environmental changes.

Overall, under straw return, shallow buried drip irrigation and mulched drip irrigation appeared to be more conducive to the accumulation of soil carbon components and the enhancement of certain carbon transformation-related enzyme activities, whereas flood irrigation exhibited a relatively weaker effect on soil carbon accumulation. Therefore, selecting appropriate irrigation techniques under straw return can not only enhance the soil carbon sequestration potential, but also improve soil nutrient supply capacity, thereby supporting the sustainable development of agricultural ecosystems.

### 4.2 Differential responses of soil microbial community composition and structure to different irrigation techniques under straw return

The results showed that the composition, structure, and network properties of soil microbial communities differed significantly among irrigation techniques under straw return. Compared with the BICS and SDCS treatments, the DPCS treatment increased the bacterial Chao1 index by 8.36 and 8.77%, respectively, whereas the fungal Chao1 index decreased significantly by 37.24 and 39.44%, respectively. Previous studies in mulched rice systems have shown that the moist conditions created by mulching can substantially increase soil particle wettability. Such moisture conditions are more favorable for bacterial proliferation, as bacteria generally respond rapidly to moist environments. The reduced fungal richness under the DPCS treatment may be associated with resource competition effects resulting from the rapid enrichment of bacterial communities. Mulched drip irrigation combined with straw return may promote the accumulation and transformation of readily available carbon substrates, enabling bacteria to gain a competitive advantage in carbon substrate utilization. This competitive dominance may suppress the proliferation of certain fungal taxa, ultimately leading to reduced fungal richness (Wang C. et al., 2025; Shabana et al., 2021; Wu Y. et al., 2026). The irrigation techniques affected soil microbial community structure in different ways. The bacterial community showed more pronounced changes under the DPCS treatment, whereas the fungal community exhibited greater structural differentiation under the SDCS treatment. The effects of the DPCS treatment on bacterial communities may be associated with the preservation and transformation of readily available carbon substrates under mulched drip irrigation. Because bacteria generally respond rapidly to changes in labile carbon substrates, the bacterial community structure exhibited more pronounced shifts under the DPCS treatment. In contrast, changes in fungal community structure were more evident under the SDCS treatment, possibly because the shallow subsurface drip lines with slight soil coverage were located closer to the surface straw decomposition zone. This condition may more strongly influence fungal taxa involved in hyphal expansion and complex organic matter decomposition, thereby resulting in greater variation in fungal community composition (Zhang B. et al., 2024).

The SDCS treatment exhibited the highest relative abundances of Proteobacteria and Chytridiomycota. Previous studies have shown that a uniform and stable water supply can create favorable growth conditions for microorganisms, particularly Proteobacteria and Chytridiomycota (Kou et al., 2025; Bai et al., 2018). Under the DPCS treatment, Mortierellomycota showed the highest relative abundance (14.61%). As a carbon-associated fungal group, Mortierellomycota may have benefited from the relatively high level of soil carbon accumulation under the DPCS treatment. In contrast, Ascomycota exhibited higher relative abundance under the BICS treatment, possibly because this group is more adaptable to environments with greater soil moisture fluctuations (Guo et al., 2022).

From the perspective of microbial life-history strategies, the R/K framework reflects the adaptive responses of microbial communities to resource availability and environmental changes, and provides an important ecological perspective for understanding microbially mediated carbon transformation and nutrient cycling processes (Sun and Han, 2025). In general, R-strategist microorganisms exhibit strong capacities for rapid resource utilization and tend to proliferate quickly under conditions with abundant readily available substrates. In contrast, K-strategist microorganisms generally grow more slowly but possess stronger competitive abilities and greater environmental tolerance, which are conducive to maintaining relatively stable resource utilization processes (Kang et al., 2026). In the present study, the DPCS treatment exhibited the highest proportion of r-strategist microorganisms, reaching 72.28%, indicating that mulched drip irrigation combined with straw return may favor the enrichment of microorganisms characterized by rapid resource acquisition. Combined with the higher SOC, EOC, and S-SC activity observed under the DPCS treatment, these results suggest that this treatment promoted the accumulation and transformation of labile carbon substrates, thereby providing a favorable substrate basis for the rapid proliferation of r-strategist microorganisms. By contrast, the SDCS treatment showed a relatively higher proportion of K-strategist microorganisms (31.28%), suggesting that shallow buried drip irrigation combined with straw return may be more conducive to the maintenance of resource-conservative microbial taxa. Together with the higher S-SL activity observed under the SDCS treatment, this finding implies that this treatment enhanced the enzymatic decomposition of complex straw-derived organic substrates, thereby shifting the microbial community toward a slower but more sustained resource utilization strategy.

Analysis of molecular ecological network patterns showed that bacterial and fungal communities responded differently to irrigation techniques. Under the BICS treatment, the bacterial network exhibited the highest number of connections. In contrast, the fungal network showed the highest connectivity under the DPCS treatment. A higher number of network connections generally indicates more complex co-occurrence relationships among community members, which may reflect a more active microbial response to changes in resource availability and environmental conditions under that treatment. Network cohesion analysis showed that the SDCS treatment exhibited the strongest negative cohesion, indicating a more stable molecular ecological network structure. Enhanced negative cohesion generally indicates stronger mutual constraints or niche differentiation within the community. Such interactions can reduce the risk of excessive dominance by a few taxa, thereby contributing to the stability of the microbial network structure. A more stable microbial network may help sustain the continuity of soil nutrient supply, thereby providing biological support for crop root nutrient uptake and yield formation (Long et al., 2025). The water supply pattern to the root zone regulated moisture infiltration and maintained a more balanced soil moisture condition. This may have enhanced microbial cooperation and reduced competitive interactions, thereby generating a stronger negative cohesion network. Such changes may also have contributed to greater stability of the microbial community structure (Affortit et al., 2024). This may also be attributed to the relatively higher proportion of K-strategist microorganisms and the more active transformation of complex carbon substrates under this treatment, which could promote stronger resource partitioning and mutual constraints among microbial taxa, thereby contributing to the stability of the network structure. Overall, irrigation techniques significantly influenced the composition and structure of soil microbial communities.

### 4.3 Differential yield-enhancing effects of irrigation techniques under straw return

This study found that, under straw return, different irrigation techniques significantly influenced maize yield through distinct water supply patterns. Water supply patterns strongly influenced soil microbial community composition, carbon transformation-related enzyme activities, and soil carbon accumulation. Under the BICS treatment, continuous water input combined with large moisture fluctuations required microbial communities to rapidly adapt to changing environmental conditions. This may have intensified both competitive and cooperative interactions, thereby shaping overall microbial network interactions and stability. The microbial community significantly affected soil carbon-related components (path coefficient = 0.9291, *p* < 0.05), which subsequently influenced maize yield (path coefficient = 0.9877, *p* < 0.001).

Under the SDCS treatment, precise and uniform water supply promoted microbial growth and reproduction (Malinowski et al., 2024). The relatively high proportion of K-strategist microorganisms (31.28%) may indicate that the environmental conditions under SDCS were favorable for resource-conservative microbial taxa. Under relatively stable moisture conditions, these microorganisms may sustain long-term soil carbon accumulation through slower growth and more efficient resource utilization. The high S-SL activity observed under the SDCS treatment (path coefficient = 0.3417, *p* < 0.05) may have enhanced straw decomposition, thereby generating more available carbon substrates and promoting soil carbon transformation and accumulation (Li Y. et al., 2025). The microbial community also strongly contributed to POC accumulation under SDCS (path coefficient = 0.8290, *p* < 0.01). In addition, S-SL and S-PPO activity was closely associated with POC accumulation (Hu et al., 2024), suggesting a potential role in maintaining long-term soil carbon stability. The microbial community further contributed to maize yield improvement (path coefficient = 0.7137, *p* < 0.01). Overall, these findings suggest that continuous and precise water supply under the SDCS treatment created a more stable microbial environment, thereby promoting soil carbon accumulation and enhancing maize yield.

The DPCS treatment significantly enhanced S-SL and S-SC activity (path coefficient = 0.9313, *p* < 0.001) and microbial community diversity (path coefficient = 0.9243, *p* < 0.001), thereby promoting organic matter decomposition and carbon transformation processes (Zhao L. et al., 2025). Under the DPCS treatment, significant EOC accumulation further improved soil fertility and promoted maize yield (path coefficient = 0.9243, *p* < 0.001). In addition, the microbial community, carbon transformation-related enzymes, and soil carbon components formed a positive feedback relationship under the DPCS treatment, which contributed to maize yield improvement.

In summary, different irrigation techniques influenced soil carbon accumulation and crop yield formation by altering soil microbial communities and the activities of carbon transformation-related enzymes. Optimizing irrigation management and selecting irrigation techniques adapted to regional conditions may effectively enhance soil carbon accumulation and crop productivity, thereby providing an efficient management strategy for sustainable agricultural development.

Building on these findings, future studies could extend the investigation in several key directions. First, the experimental design focuses exclusively on the 0–20 cm plow layer and does not encompass deeper soil horizons. Future studies will extend nutrient monitoring to deeper soil layers, enabling a more comprehensive evaluation of responses across the entire soil profile. Second, microbial characterization in this study relies solely on amplicon sequencing, which provides information on relative taxonomic abundance but is limited in sequencing depth, breadth, and insight into functional potential. Increasing sequencing depth and integrating multi-omics approaches will facilitate a more comprehensive characterization of microbial diversity and metabolic functions. Third, the current analyses are primarily based on correlation and statistical modeling, and experimental validation will be required to further substantiate the inferred relationships. Finally, measurements of enzyme activities and microbial communities are obtained at only a limited number of time points and therefore do not fully capture temporal fluctuations in the soil microenvironment. Long-term dynamic sampling across multiple growing seasons, combined with real-time monitoring of microenvironmental conditions, will help further validate and deepen our understanding of the underlying interaction mechanisms.

## 5 Conclusion

Different irrigation techniques under straw return significantly affected maize yield, soil carbon components, enzyme activities, and microbial communities. The results showed that the overall effect of the BICS treatment on maize yield promotion was relatively limited. In contrast, the SDCS treatment was characterized by higher POC content, greater S-SL activity, and increased fungal richness, together with more pronounced fungal community differentiation, a higher proportion of K-strategist microorganisms, and a more stable microbial network structure. The DPCS treatment exhibited higher maize yield, soil carbon content, S-SC activity, bacterial richness, and greater community differentiation. Regarding yield formation, under the BICS treatment, changes in soil carbon components were strongly associated with variations in microbial communities, which were further linked to maize yield variation, with microbial network structure and DOC identified as key associated factors. Under the SDCS treatment, strong synergistic relationships were observed among soil carbon components, microbial communities, and carbon transformation-related enzyme activities, among which carbon components and microbial communities were closely associated with maize yield improvement. In particular, shifts in microbial life-history strategies and POC accumulation appeared to be important influencing factors. Under the DPCS treatment, higher microbial diversity, enhanced carbon transformation-related enzyme activities, and increased EOC accumulation were strongly correlated with maize yield enhancement. Overall, this study demonstrates that under straw return conditions, irrigation techniques influence soil carbon sequestration and the maintenance of crop yield in non-rainfed croplands. Nevertheless, further research incorporating deeper soil profiles, multi-omics approaches, and long-term, site-specific monitoring will be required to validate these findings and deepen mechanistic understanding of these interactions.

## Data Availability

The datasets presented in this study can be found in online repositories. The names of the repository/repositories and accession number(s) can be found at: https://www.ncbi.nlm.nih.gov/, PRJNA1417113.

## Footnotes

1
http://rdp.cme.msu.edu/
