# Supplementary material for: Effects of irrigation techniques on soil carbon sequestration and yield maintenance in non-rainfed croplands under straw return
Source: Front Microbiol. 2026 Jun 26;17:1844595. doi: 10.3389/fmicb.2026.1844595 (PMC13350467; doi:10.3389/fmicb.2026.1844595)
Supplement: Supplementary file 1 [file Data_Sheet_1.docx]

**Effects of irrigation techniques on soil carbon sequestration and yield maintenance in non-rainfed croplands under straw return**

Wei Cheng ^1^, Juntao Cui ^2^, Bing Zhang ^2*^ and Long Ming ^3*^

^1^College of Humanities & Information, Changchun University of Technology, Changchun, China. ^2^College of Resources and Environmental, Jilin Agricultural University, Changchun, China. ^3^College of Landscape Architecture, Changchun University, Changchun, China.

*Correspondence: Bing Zhang and Long Ming

Email: zb18686446806@126.com; minglong8788@163.com

**Supplementary figure**


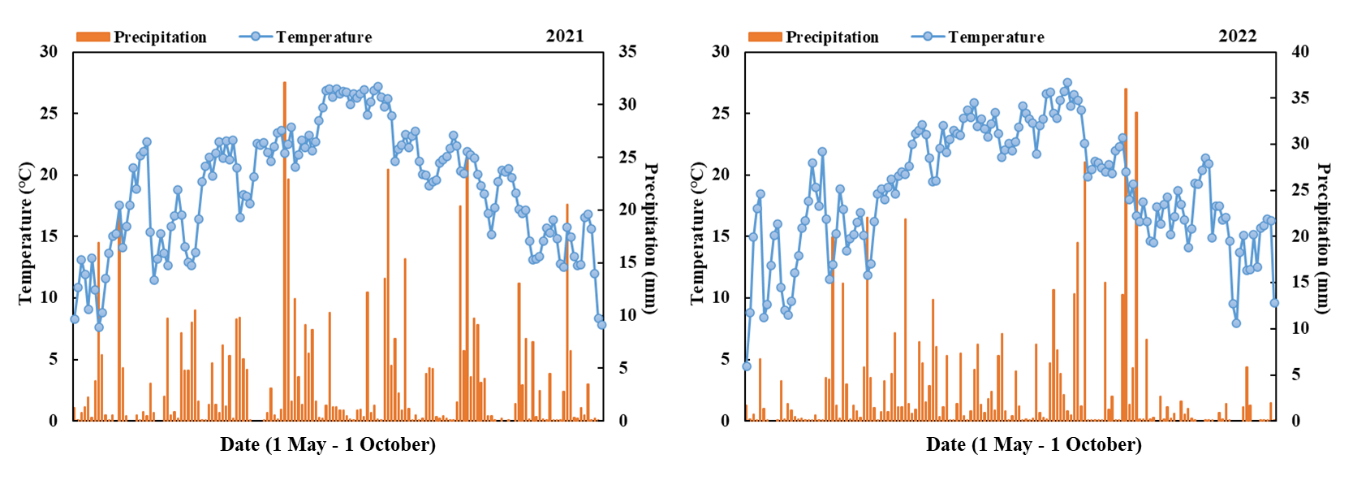


**Fig.S1** Meteorological conditions during two consecutive maize growing seasons


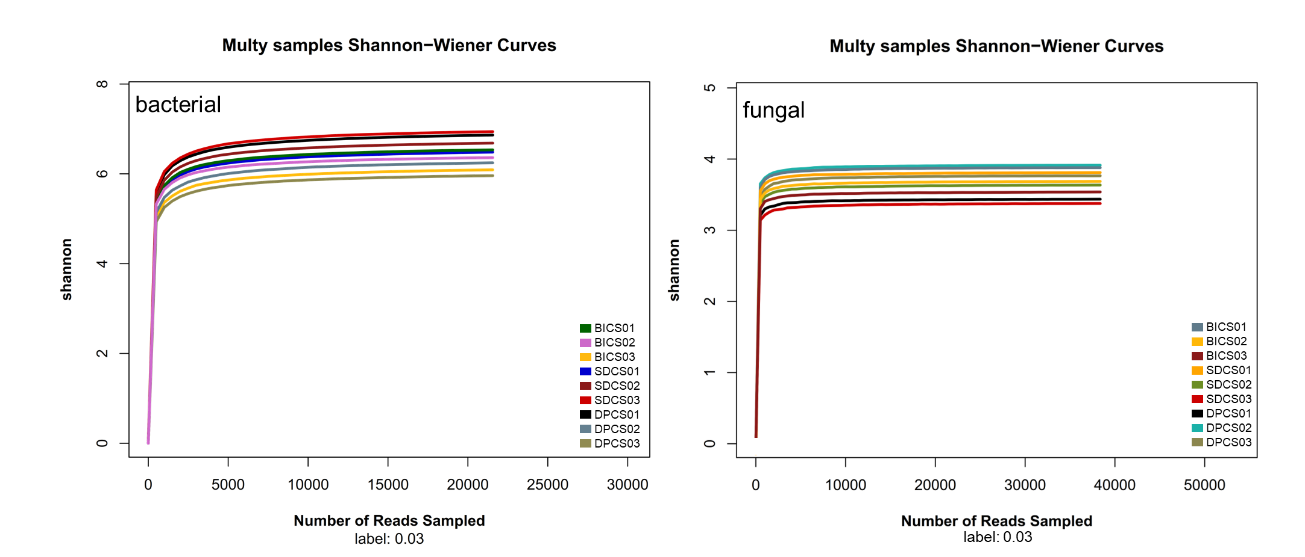


**Fig.S2** Rarefaction curves for bacterial and fungal sequencing datasets

**Supplementary tables**

**Table S1** The present study defines copiotrophic and oligotrophic microbial groups based on an extensive literature review

| Phylum | Trophic lifestyle | Reference |
| --- | --- | --- |
| Firmicutes | Copiotroph | Chen et al., 2022; Francioli et al., 2016; Li et al., 2021; Stone et al., 2023; Nemergut et al., 2010 |
| Bacteroidetes | Copiotroph | Chen et al., 2022; Fierer et al., 2007; Fierer et al., 2012; Männistö et al., 2016; Stone et al., 2023; Zhou et al., 2017; Padmanabhan et al., 2003; Li et al., 2021 |
| Proteobacteria | Copiotroph | Chen et al., 2022; Li et al., 2021; Stone et al., 2023; Padmanabhan et al., 2003; Cleveland et al., 2007; Fierer et al., 2012; Hungate et al., 2015; Leff et al., 2015; |
| Ascomycota | Copiotroph | Ho et al., 2017; Yao et al., 2017; Li et al., 2021 |
| Actinobacteria | Oligotroph | Li et al., 2021; Zhou et al., 2017 |
| Acidobacteria | Oligotroph | Cleveland et al., 2007; Hungate et al., 2015; Leff et al., 2015; Chen et al., 2022; Fierer et al., 2007; Fierer et al., 2012; Li et al., 2021; Männistö et al., 2016; Stone et al., 2023; Zhou et al., 2017 |
| Verrucomicrobia | Oligotroph | Bergmann et al., 2011; Chen et al., 2022; Männistö et al., 2016; Stone et al., 2023; Fierer et al., 2012; |
| Planctomycetes | Oligotroph | Li et al., 2021; Männistö et al., 2016; Stone et al., 2023 |
| Gemmatimonadetes | Oligotroph | Chen et al., 2022; Li et al., 2021 |
| Chloroflexi | Oligotroph | Chen et al., 2022; Li et al., 2021; Stone et al., 2023; Fierer et al., 2012 |

**Table S2** NCBI Metagenome Environmental Information Table

**Table S3** NCBI SRA_Metadata Table

References

Bernhardt-Römermann M, Baeten L, Craven D, et al. (2015) Drivers of temporal changes in temperate forest plant diversity vary across spatial scales. Global Change Biol. 21(10):3726-3737.

Chen H, Jing Q, Liu X, et al. (2022) Microbial respiratory thermal adaptation is regulated by r-/K-strategy dominance. Ecology Lett. 25(11):2489-2499.

Cleveland CC, Nemergut DR, Schmidt SK, et al. (2007) Increases in soil respiration following labile carbon additions linked to rapid shifts in soil microbial community composition. Biogeochemistry 82:229-240.

Fierer N, Bradford MA, Jackson RB (2007) Toward an ecological classification of soil bacteria. Ecology 88(6):1354-1364.

Fierer N, Lauber CL, Ramirez KS, et al. (2012) Comparative metagenomic, phylogenetic and physiological analyses of soil microbial communities across nitrogen gradients. The ISME Journal 6(5):1007-1017.

Francioli D, Schulz E, Lentendu G, et al. (2016) Mineral vs. Organic Amendments: Microbial Community Structure, Activity and Abundance of Agriculturally Relevant Microbes Are Driven by Long-Term Fertilization Strategies. Front. Microbiol. 7:1446.

Ho A, Lonardo D, Bodelier DP (2017) Revisiting life strategy concepts in environmental microbial ecology. FEMS Microbiol. Ecol. 93:3.

Hungate BA, Mau RL, Schwartz E, et al. (2015) Quantitative microbial ecology through stable isotope probing. Appl. Environ. Microb. 81:7570-7581.

Leff JW, Jones SE, Prober SM, et al. (2015) Consistent responses of soil microbial communities to elevated nutrient inputs in grasslands across the globe. P. Natl. Acad. Sci. Usa. 112:10967-10972.

Li H, Yang S, Semenov MV, et al. (2021) Temperature sensitivity of SOM decomposition is linked with a K-selected microbial community. Global Change Biol. 27:2763-2779.

Männistö M, Ganzert L, Tiirola M, et al. (2016) Do shifts in life strategies explain microbial community responses to increasing nitrogen in tundra soil? Soil Biol. Biochem. 96:216-228.

Nemergut DR, Cleveland CC, Wieder WR, et al. (2010) Plot-scale manipulations of organic matter inputs to soils correlate with shifts in microbial community composition in a lowland tropical rain forest. Soil Biol. Biochem. 42:2153-2160.

Padmanabhan P, Padmanabhan S, DeRito C, et al. (2003) Respiration of 13C-labeled substrates added to soil in the field and subsequent 16S rRNA gene analysis of 13C-labeled soil DNA. Appl. Environ. Microb. 69:1614-1622.

Stone BWG, Dijkstra P, Finley BK, et al. (2023) Life history strategies among soil bacteria-dichotomy for few, continuum for many. The ISME Journal 17(4):611-619.

Yao F, Yang S, Wang Z, et al. (2017) Microbial taxa distribution is associated with ecological trophic cascades along an elevation gradient. Front. Microbiol. 8:2071.

Zhou ZH, Wang CK, Jiang LF, et al. (2017) Trends in soil microbial communities during secondary succession. Soil Biol. Biochem. 115:92-99.
